# Cellular Expression, Trafficking, and Function of Two Isoforms of Human ULBP5/RAET1G

**DOI:** 10.1371/journal.pone.0004503

**Published:** 2009-02-18

**Authors:** Robert A. Eagle, Gillian Flack, Anthony Warford, Jesús Martínez-Borra, Insiya Jafferji, James A. Traherne, Maki Ohashi, Louise H. Boyle, Alexander D. Barrow, Sophie Caillat-Zucman, Neil T. Young, John Trowsdale

**Affiliations:** 1 Cambridge Institute for Medical Research, Wellcome Trust/MRC Building, Addenbrookes Hospital, Cambridge, United Kingdom; 2 Atlas of Protein Expression Group, Wellcome Trust Sanger Institute, Wellcome Trust Genome Campus, Hinxton, Cambridgeshire, United Kingdom; 3 INSERM U561 AVENIR Team, Hôpital St-Vincent de Paul, Paris, France; 4 Department of Pathology, University of Cambridge, Cambridge, United Kingdom; 5 California Institute of Technology, MC170-25, Pasadena, California, United States of America; Centre de Recherche Public de la Santé (CRP-Santé), Luxembourg

## Abstract

**Background:**

The activating immunoreceptor NKG2D is expressed on Natural Killer (NK) cells and subsets of T cells. NKG2D contributes to anti-tumour and anti-viral immune responses *in vitro* and *in vivo*. The ligands for NKG2D in humans are diverse proteins of the MIC and ULBP/RAET families that are upregulated on the surface of virally infected cells and tumours. Two splicing variants of ULBP5/RAET1G have been cloned previously, but not extensively characterised.

**Methodology/Principal Findings:**

We pursue a number of approaches to characterise the expression, trafficking, and function of the two isoforms of ULBP5/RAET1G. We show that both transcripts are frequently expressed in cell lines derived from epithelial cancers, and in primary breast cancers. The full-length transcript, RAET1G1, is predicted to encode a molecule with transmembrane and cytoplasmic domains that are unique amongst NKG2D ligands. Using specific anti-RAET1G1 antiserum to stain tissue microarrays we show that RAET1G1 expression is highly restricted in normal tissues. RAET1G1 was expressed at a low level in normal gastrointestinal epithelial cells in a similar pattern to MICA. Both RAET1G1 and MICA showed increased expression in the gut of patients with celiac disease. In contrast to healthy tissues the RAET1G1 antiserum stained a wide variety or different primary tumour sections. Both endogenously expressed and transfected RAET1G1 was mainly found inside the cell, with a minority of the protein reaching the cell surface. Conversely the truncated splicing variant of RAET1G2 was shown to encode a soluble molecule that could be secreted from cells. Secreted RAET1G2 was shown to downregulate NKG2D receptor expression on NK cells and hence may represent a novel tumour immune evasion strategy.

**Conclusions/Significance:**

We demonstrate that the expression patterns of ULBP5RAET1G are very similar to the well-characterised NKG2D ligand, MICA. However the two isoforms of ULBP5/RAET1G have very different cellular localisations that are likely to reflect unique functionality.

## Introduction

The activating immunoreceptor NKG2D has been established as an important component of the innate immune system. In humans NKG2D is constitutively expressed on NK cells, γδ T cells, and CD8^+^ T cells[1]. NKG2D is not normally found on CD4^+^ T cells, however it can be expressed in these cells in patients with rheumatoid arthritis[2], cancer[3], and when stimulated with HCMV[4]. NKG2D can invoke powerful immune responses against target cells expressing its ligands both *in vitro* and *in vivo* [1], [5], however cellular activation is likely to require synergy with other signals delivered by cytokines or other receptors[6], [7], [8].

The ligands of NKG2D are surprisingly diverse proteins that are structurally related to MHC class I[9], [10]. In humans they comprise two members of the MIC family and five members of the ULBP/RAET family[11], [12], [13], [14]. According to the paradigm of NKG2D mediated immune recognition, ligands for the receptor are expressed on cells subject to stress, for example cells infected with viruses such as HCMV, or during tumourigenesis[15], [16]. The importance of NKG2D in viral infection is evidenced by the fact that both human and murine cytomegalovirus encode immune evasion molecules designed to prevent cell surface expression of NKG2D ligands[15].

NKG2D ligands are widely expressed on cancer cells. Activation of DNA damage pathways during tumour development has been reported to be a central mechanism leading to NKG2D ligand expression [17]. There is evidence that NKG2D participates in a process of immunosurveillance for cancer. In mice, the ectopic expression of NKG2D ligands mediates immune responses to transplanted tumours[18], [19]. Neutralising NKG2D with antibodies decreases host protection to experimentally induced tumours[20] and an NKG2D knockout mouse showed an increased susceptibility to some model cancers [21]. Therefore it is proposed that in order to progress, cancers must evade NKG2D mediated immune responses. One evasion mechanism may be the production of TGF-β by tumours, which downregulates NKG2D expression[22], [23]. NKG2D may also be downregulated by soluble ligands released from the surface of cancer cells[24], and by chronic exposure to cells expressing its ligands[25], [26], [27]. These studies suggest that NKG2D mediated immunity may become anergised during tumour development.

The MICA molecule has restricted expression in normal tissues but is found in normal gut epithelial cells[28]. In healthy individuals MICA seems to be largely inside the cell, however in celiac disease (CD) MICA expression is increased and redistributes to the cell surface where it can provoke autoimmune attack and villous atrophy[29], [30]. Inappropriate NKG2D ligand expression has a role in other autoimmune diseases, including Crohn's disease[31], autoimmune diabetes[32], and rheumatoid arthritis[28].

An open question in the field is why are their so many ligands for the same receptor? Intriguingly NKG2D ligands possess markedly different domain structures. ULBP1-3 molecules are GPI anchored to the cell surface, whilst MICA/B, ULBP4/RAET1E, and ULBP5/RAET1G have transmembrane and cytoplasmic domains of variable length and sequence[12], [13], [14], [33]. NKG2D ligands are also capable of being expressed independently of each other[12], [34], [35]. This suggests that NKG2D ligands may not all be functionally equivalent. The predominant driver of NKG2D ligand diversity is proposed to be an immunological arms race with viruses, which have in turn acquired a varied array of immunoevasin molecules that prevent NKG2D ligands being expressed at the cell surface[10]. However this is not the whole story. NKG2D ligands have clearly evolved other unique properties that do not relate to their susceptibility to viral immunoevasins. One example is MICA, which possesses motifs that allow it to be targeted to specific membranes in specialised epithelial cell layers[36]. Therefore it is essential to examine the expression profile and properties of individual MIC and ULBP/RAET molecules to get a fuller understanding of NKG2D function.

Two alternative transcripts of ULBP5/RAET1G have previously been identified (named RAET1G1 and RAET1G2)[14], [37]. Here we examine the expression, trafficking and function of each variant in detail.

## Materials and Methods

### Antibody generation and western blotting

Polyclonal antibody to RAET1G was raised in rabbits using two peptides corresponding to part of the CYT of the protein. The peptides were:

CNNGAARYSEPLQVSIS and CSHGHHPQSLQPPPHPP

Peptides were manufactured and coupled to Ovalbumin by Southampton Polypeptides. The antiserum was raised in rabbit using a combination of both peptides, separately coupled to ovalbumin, by Harlan Seralabs. The polyclonal antibody was purified by the caprylic acid/ ammonium sulphate precipitation method. For western blotting, ∼5*10^5^ cells were lysed straight into reducing SDS-PAGE buffer, boiled and separated by SDS-PAGE. For soluble RAET1G detection in tissue culture 40 µl tissue culture medium was boiled with SDS-PAGE buffer and loaded onto a gel. Western blotting was carried out according to standard protocols. Membranes were probed with either anti-RAET1G polyclonal, rabbit polyclonal anti-V5 (Invitrogen), or mouse monoclonal anti-β-actin (Sigma) followed by species specific HRP-conjugated secondary antibodies (Dako).

### Cell lines, and primary cell cultures

Cell lines are as described previously[14]. Primary NK cells were isolated from peripheral blood using standard Ficoll isolation of mononuclear cells, followed by use of an NK cell negative isolation kit (Dynal) to deplete unwanted cell populations. NK cells were cultured in RPMI-1640 medium containing penicillin, streptomycin, 10% human serum and 100 U/ml rIL-2. Prior to use, isolated NK cells were shown to be CD56^+^, NKG2D^+^ and CD3^−^ (data not shown).

### RT-PCR

Cell line and primary breast cancer mRNA was a gift from Dr Cherie Blenkiron and Prof Carlos Caldas[38]. Fresh frozen primary tumours were collected with consent from patients between 1997–2000 by the Addenbrooke's Hospital Histology Tissue Bank, Cambridge, UK. All samples were used with appropriate Local Research Ethics Committee approval, Addenbrookes Hospital, and were collected and stored with written consent from patients. First strand cDNA synthesis from cell line and primary tumour mRNAs was performed using the Superscript-III cDNA Synthesis Kit (Invitrogen). cDNA was quantitated and adjusted so that 200 ng was used in each PCR reaction. Splice form specific primers were RAET1G1Fwd: CCATGTCCTCAGGCACAGC; RAET1G1Rev: CAGGGAACCATCAAGATATGG; RAET1G2Fwd: AGCCCCGCGTTCCTTCTA; RAET1G2Rev: GGGTCAGACTGTGCCTCCT. PCR reactions were carried out for 35 cycles with an annealing temperature of 62°C on a Peltier Thermal Cycler.

### Constructs and cDNA expression in mammalian cells and bacteria

An N-terminal GFP fusion construct of RAET1G1 and ULBP2 was created in the vector PK1 [39]. The resulting construct contained a leader peptide followed by GFP, a Myc tag, and a short linker peptide fused to the relevant cDNA. Stable cell lines in HT1080 cells were created with a lentiviral exression system (kind gift of Dr Paul Lehner, [40]). C-terminal V5 tagged constructs were created using the pEF6/V5-His TOPO TA Expression Kit (Invitrogen). IMAGE clones 3070730 and 2911855 were used as templates for RAET1G1 and RAET1G2 constructs respectively. For western blot, ∼5*10^5^ Cos7 cells were transiently transfected with 1 µg PK1-RAET1G1 plasmid using Fugene reagent (Roche). For expression of V5 tagged constructs 5*10^6^ Cos7 cells were transfected with 5 µg plasmid, again with Fugene, and cells were harvested for western blot and supernatant collected for NK cell assays. Soluble recombinant RAET1G extracellular domain was produced as a six histidine N-terminal fusion protein using a plasmid described previously[14]. The protein was expressed in inclusion bodies in BL21 *E.coli* (Novagen) and solubilised in 6 M guanidine hydrochloride. Refolding was achieved by a stepwise dilution into a Tris and Arginine containing buffer followed by concentration by centriprep (Amicon). Prior to use in NK cell assays recombinant RAET1G was dialysed into PBS.

### Confocal microscopy

Confocal microscopy was carried out as described previoulsy^33^. For staining mouse monoclonal antibodies to MHC class I (W6/32, kind gift from Dr Adrian Kelly), GM130 (BD Transduction Labs), EEA1 (BD Transduction Labs) were used followed by anti-mouse Alexa Fluor 568 secondary antibody (Molecular Probes). Rabbit polyclonal anti-RAET1G or anti-calreticulin (BD Transduction Labs) was used followed by anti-rabbit Alexa Fluor 568. For antibody internalisation experiments transfected cells were incubated with rabbit anti-myc antibody (AbCam) for 30 min at 4°C and then warmed to 37°C for 2 hours prior to fixing and staining with secondary antibody

### Flow Cytometry

HT1080 cell lines stably expressing PK1-ULBP2 and PK1-RAET1G were stained with anti-GFP monoclonal antibody (Roche) for 30 minutes at 4°C, followed by goat anti-mouse Alexa Fluor 633 nm conjugate (Molecular Probes). As the stable cell lines were roughly 70% GFP positive, GFP negative cells were gated out in analysis. Data was then collected using BD FACSCalibur. For primary NK cell analysis cells were cultured for 2 days either in the presence or absence of soluble RAET1G and then analysed for NKG2D expression with a PE conjugated anti-NKG2D mAb (Coulter).

### Radiolabelling and immunoprecipitation

Radiolabelling and immunoprecipitations were carried out as described previously [41]. Briefly, 10^7^ HT1080 stable cell lines were starved in methionine/cysteine free tissue culture media (Sigma) and then labelled with 1 mCi [^35^S] methionine and cysteine Pro-mix (Amersham) for 10 minutes at 37°C. The cells were then chased for 180 minutes in media containing unlabelled methionine/cysteine media. After washing in PBS radiolabelled cells were lysed in 1% Triton X-100 lysis buffer (150 mM NaCl, 20 mM Tris, 1 mM EDTA, 5 mM MgCl_2_ with protease inhibitors from Roche). After removing nuclei and debris by centrifugation 1 µg anti-GFP monoclonal antibody (Roche) was added to immunoprecipitate GFP fusion proteins, and protein A sepaharose (Amersham) added to bind immune complexes. After washing the bound proteins were eluted in reducing SDS-PAGE buffer and half the eluate subject to digestion with Endo Hf (New England Biolabs).

### Immunohistochemistry

Immunohistochemistry was undertaken on paraffin wax tissue microarrays[42], [43]. The first was prepared using guided tissue selection transferring 2×0.6 mm diameter cores from each formalin fixed donor tissue into the recipient array. The normal tissue microarray contained a total of 344 cores from 172 donor samples as listed in Table 1. A similar array comprising normal and tumour tissues was probed with purified pre-immune serum in equal amounts as a control for staining, All samples were obtained from Medical Solutions plc with ethical approval obtained from LREC, Cambridge, UK. To evaluate the tissue distribution of the RAET1G antibody in tumours, sections of commercial paraformaldehyde fixed tissue microarray (Petagen Inc, code A201(1)) containing 1 mm cores from 35 epithelial cancer samples (Table 1) were also immunostained. Automated immunohistochemistry was undertaken using a Ventana Medical Systems Discovery™ system. Sections were dewaxed, pre-treated with mild cell conditioner 1 (Tris borate/EDTA, pH 8.0) then incubated in the rabbit anti RAET1G antibody at 10 µg/ml for 20 min at 37°C. Staining was optimised by staining with a range of antibody concentrations 10 µg/ml was selected as the optimum concentration. For detection an avidin/biotin block preceded application of biotinylated goat anti rabbit (DakoCytomation code E0432) diluted 1/100 for 8 min at 37°C. The biotinylated antibody was then detected using a streptavidin/biotin/peroxidase kit (Ventana, DAB MAP™, code 760-124). The protocol was completed by automated haematoxylin counterstaining followed by manual dehydration clearing and mounting in resinous mountant. Controls were included within each immunohistochemical runs. Anti lysozyme and vimentin antibodies were used as positive controls to verify the antigenic preservation of the tissue cores. These controls provided positive staining in all tissue cores. To establish if any staining present in the tissues was due to non-specific interaction of the detection reagents slides were also processed without the RAET1G antibody. No tissue staining was observed in these preparations. Images of the stained tissue microarray cores were automatically captured using an Ariol SL-50 automated image capture and system (Applied Imaging Inc) using a ×20 objective. Tissue sections and staining protocols for celiac disease patients are as previously published [29].

**Table 1:** Composition of Tissue Microarrays.

**Internal tissue microarray**
Normal tissues: Adrenal cortex (5), adrenal medulla (2), aorta (5), bladder (3), breast (5), cardiac muscle (5), cerebellum (5), cerebrum (5), colon (5), fallopian tube (5), ileum (5), kidney cortex(4), kidney medulla (4), liver (4), lung (5), lymph node (5), oesophagus (4), ovary (3), pancreas (5), parathyroid (1), peripheral nerve (5), pituitary (5), placenta (5), prostate (5), skin (4), spinal cord (5), spleen (5), stomach (3), striated muscle (5), testis (4), thyroid (5), tonsil (5), ureter (3), uterus,endometrium (5), uterus myometrium (5) *.
**Cancer tissue microarray**
Breast ductal cell carcinoma (2), colon adenocarcinoma (2), kidney renal cell carcinoma (2), liver cholangiocarcinoma (2), liver hepatoma (2), lung adenocarcinoma (1), lung squamous carcinoma (2), oesophagus basaloid carcimona (1), oesophagus squamous cell carcinoma (1), ovary mucinous carcinoma(2), ovary serous carcinoma (2), rectal adenocarcinoma (2), skin squamous cell carcinoma (1), stomach adenocarcinoma (1), stomach signet ring cell carcinoma (3), thyroid gland follicular carcinoma (2), thyroid gland papilliary carcinoma (2), uterus endometrioid carcinoma (2), uterus squamous cell carcinoma (2).

* Number of donor samples is shown in parenthesis.

## Results

### Expression of transcripts encoding two different isoforms of ULBP5/RAET1G

**Figure 1:**
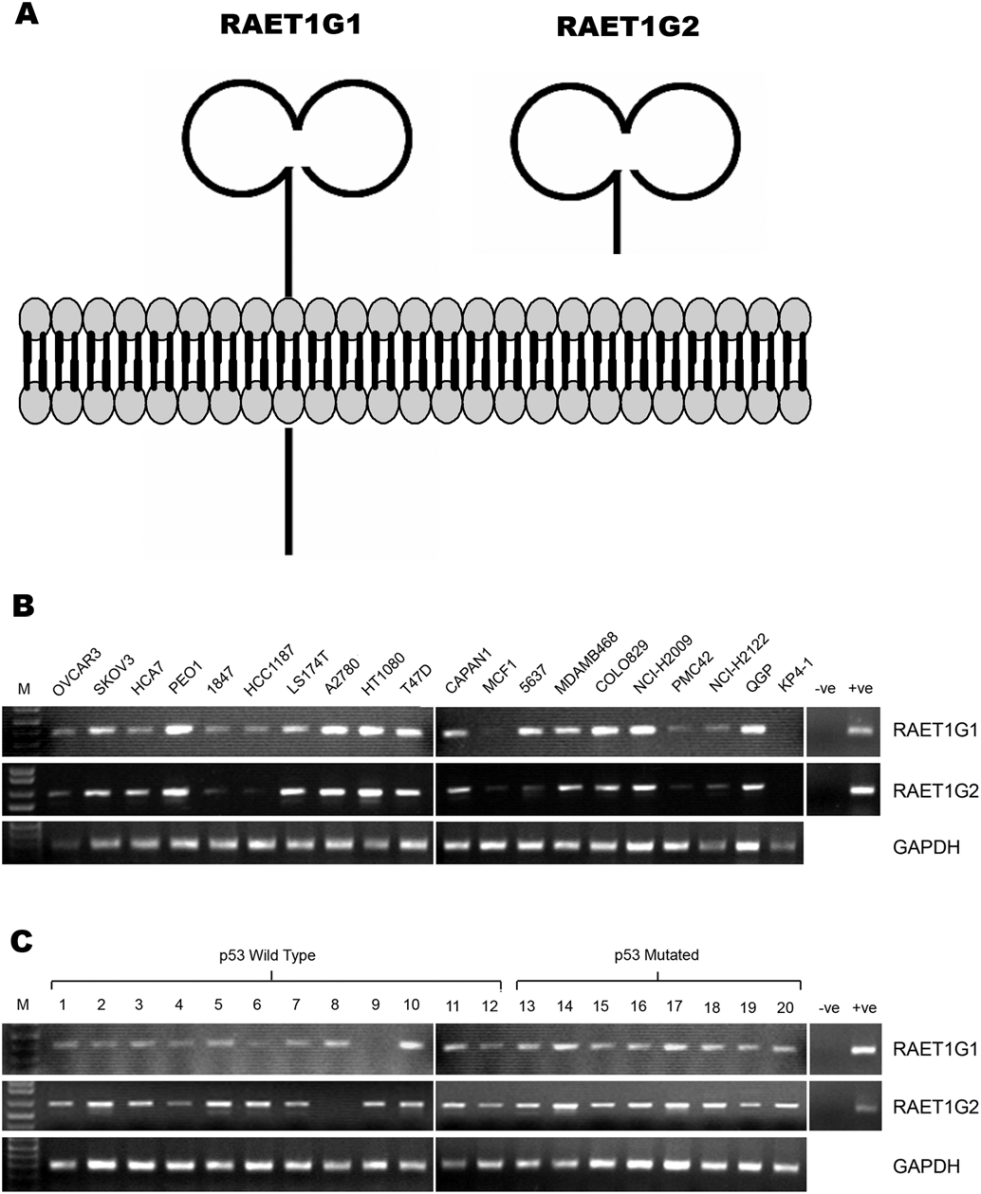
Expression of two different isoforms of ULBP5/RAET1G. (A) Two splicing variants of RAET1G have previously been identified. The full-length transcript, RAET1G1, is predicted to encode a protein with two MHC class I-related alpha domains, a transmembrane domain, and a cytoplasmic domain of 100 amino acids. RAET1G2 is predicted to encode a truncated protein that lacks the transmembrane and cytoplasmic domain. (B) Twenty epithelial cancer derived cell lines were analysed by RT-PCR for expression of full-length RAET1G1 transcript and the truncated splice variant RAET1G2. Both transcripts were present in most cell lines tested, at variable levels. (C) Expression of both variants was also very frequent in cDNA derived primary breast tumours. Expression of RAET1G1 and RAET1G2 appeared independent of p53 mutation status in a sample set of 12 tumours encoding wild-type p53 and 8 encoding mutated p53. Primers for GAPDH were used as a control for cDNA quantity.

We have previously identified two splicing variants of ULBP5/RAET1G, termed RAET1G1 and RAET1G2. The protein topology analysis programme Phobius [44] predicts that RAET1G1 encodes a protein with a transmembrane domain and a cytoplasmic domain of approximately 100 amino acids (Figure 1A). RAET1G2 has a premature stop codon before the putative transmembrane domain due to alternative splice site usage in exon 4 [14] and is predicted to encode a soluble molecule (Figure 1A). Splice form specific primers were designed to amplify each variant by RT-PCR. Specificity was tested by sequencing PCR products, and primers were designed across intron/exon boundaries in order to exclude the possibility that PCR products were amplified from contaminating genomic DNA. Both RAET1G1 and RAET1G2 were widely expressed in a panel of 20 epithelial cancer cell lines (Figure 1B). Both transcripts were also widely expressed in a panel of cDNAs derived from primary breast cancers (Figure 1C). RAET1G expression in breast cancer did not correlate with p53 mutation status, as both p53 wild-type and p53 mutated tumours frequently expressed both transcripts (Figure 1C). GAPDH primers are a described previously [35].

### Generation of anti-RAET1G antibodies

**Figure 2:**
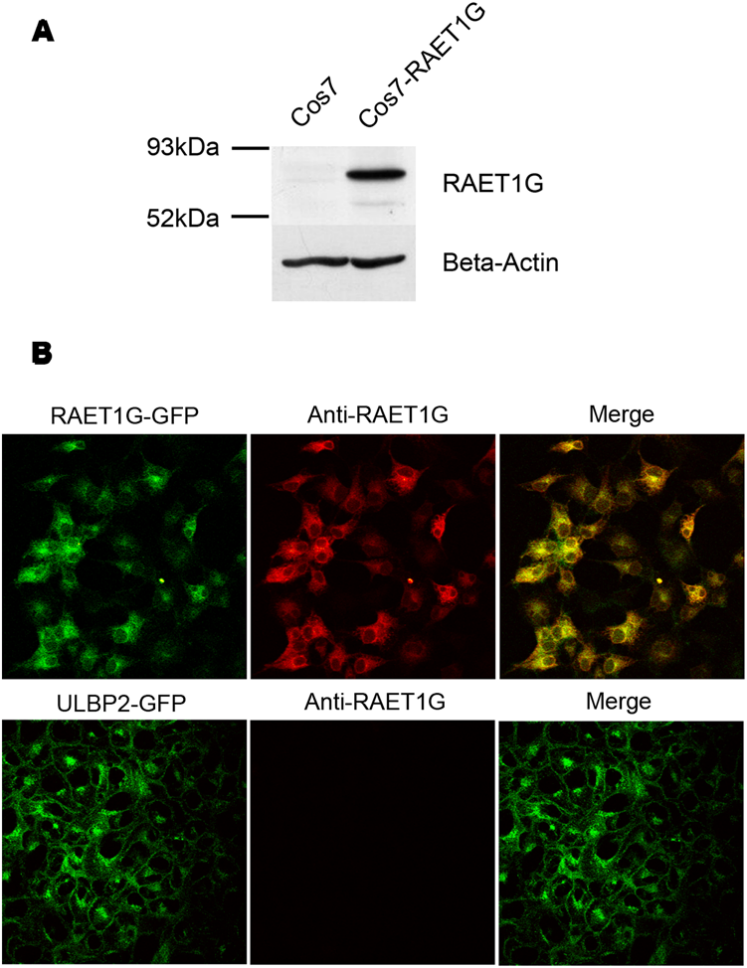
Generation of specific anti-RAET1G1 antiserum. (A) A rabbit polyclonal antiserum was raised against the cytoplasmic domain of RAET1G. By western blot a protein of approximately 70 kDa was recognised in Cos7 cells transfected with a construct encoding an RAET1G-GFP fusion protein, but not in untransfected Cos7 cells. (B) By confocal microscopy the anti-RAET1G antiserum specifically stains (red) Cos7 cells transfected with RAET1G-GFP (green), but not the highly related molecule ULBP2-GFP.

In order to investigate the cellular expression of RAET1G1 we raised specific polyclonal antiserum. The extracellular domain of RAET1G is almost identical to ULBP2, therefore we chose to generate rabbit polyclonal antiserum peptides derived from the unique cytoplasmic domain of RAET1G1. By western blotting the antiserum specifically recognised RAET1G1-GFP transfected into Cos7 cells (Figure 2A). By confocal microscopy the anti-RAET1G1 antiserum clearly stained Cos7 cells transfected with RAET1G1-GFP, but not the highly related molecule ULBP2 (Figure 2B). *ULBP2* is the most closely related gene to *RAET1G* in the human genome.

### RAET1G protein has restricted expression in normal tissues

**Figure 3:**
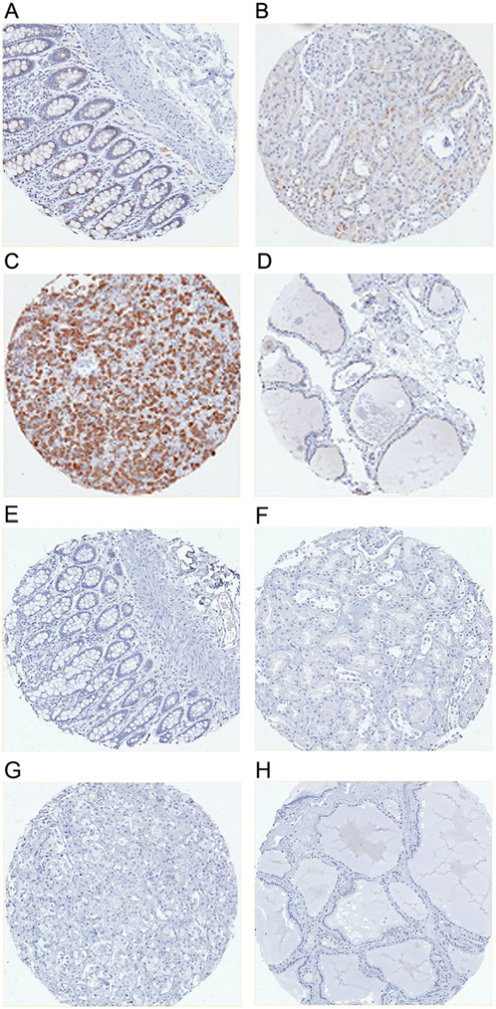
Immunohistochemical localisation of RAET1G1 in normal human tissues. The anti-RAET1G antiserum was used to stain a tissue array of 344 cores from 172 donors that represented 35 normal tissues. Staining was very infrequently observed, but was present in (A) epithelial cells of colon (B) a minority of tubular cells in kidney, (C) majority of endocrine cells of the anterior pituitary and (D) cells lining thyroid follicles. The pituitary staining may represent true RAET1G1 expression or epitope cross reactivity, as has been shown to occur with other antibodies [53], [54]. All other normal tissues were negative. Pre-immune serum did not stain any of these tissues; (E) colon, (F) kidney, (G) pituitary, and (H) thyroid.

Limited expression of the NKG2D ligand MICA in normal tissues has been previously reported [28], however no systematic study of other NKG2D ligands has been conducted. We used our RAET1G1 specific antiserum to probe a tissue microarray, comprised of 344 cores from 172 donors, representing 35 normal tissues (Table 1). Purified pre-immune rabbit serum did not stain any core. RAET1G1 antibody staining was demonstrated only in four normal tissues (Figure 3). These include gut epithelium, that has previously been shown to express RAET1G1 transcript [14], and stained with the anti-RAET1G1 antiserum in two of five individuals tested. Expression of RAET1G1 in the gut is also noteworthy as MICA has widely been reported to be constitutively expressed in the gut epithelium, and involved in gut pathology [28], [29], [31], [45].

### Expression of RAET1G and MICA in normal gut epithelium and in celiac disease

**Figure 4:**
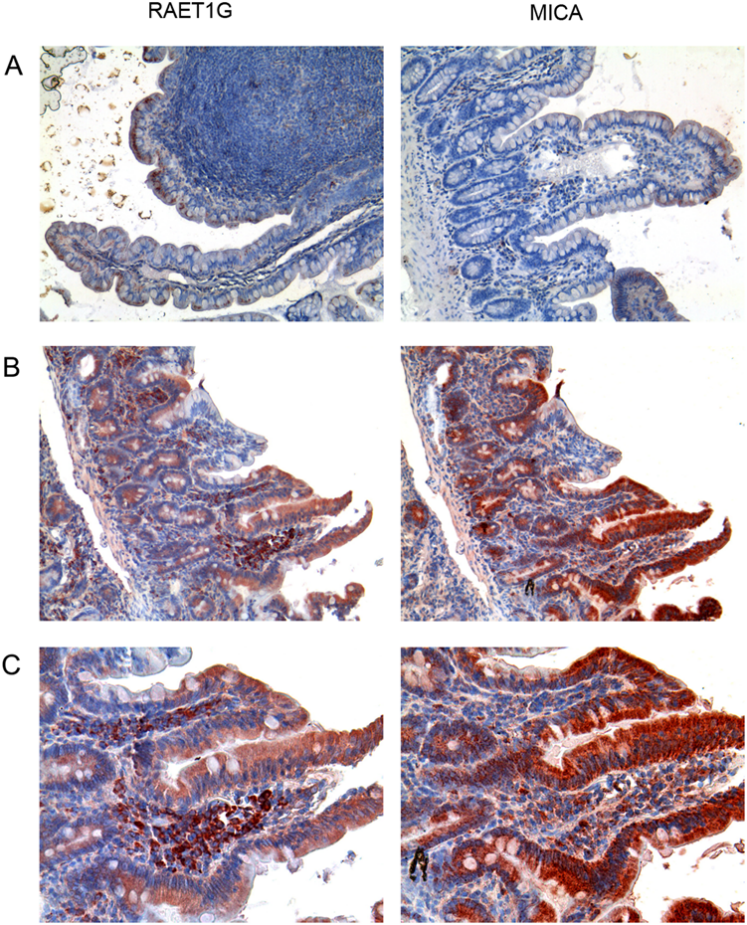
Highly similar expression of RAET1G1 and MICA in healthy gut epithelium, and in patients with celiac disease. (A) Expression of RAET1G and MICA in normal epithelial cells of the intestine. MICA was staining was with the monoclonal antibody SR99[55]. Both proteins exhibited highly similar staining patterns of granular intracytoplasmic staining in epithelial cells of the brush border but not in the crypts. The MICA staining is essentially identical to previously published data where this pattern was indicative of low cell surface expression[29]. (B) RAET1G and MICA staining in sections from the same patient with celiac disease. Both RAET1G and MICA showed increased and redistributed expression in the epithelial cell layer, with these cells exhibiting a more diffuse staining pattern. In the case of MICA this staining pattern has been shown to indicate higher cell surface expression of the protein[29]. (C) Higher magnification image of the same section. Both RAET1G and MICA show some staining of intraintestinal cells that may be plasma cells. For each antibody four healthy controls and eight celiac disease patients were stained and the data are representative of the pattern seen in all samples.

Induction of MICA expression in gut epithelial cells is known to be an important factor in the development of CD[29], [45]. We carried out a direct comparison of MICA and RAET1G expression in both normal intestine and in biopsies from patients with CD by immunohistochemistry. Both MICA and RAET1G1 antibodies gave punctate staining of villous epithelial cells that was largely intracellular in normal control sections (Figure 4A). In CD sections there was much higher intensity staining of the villous epithelium and the staining exhibited altered distribution throughout these cells (Figure 4B, 4C). In the case of MICA this staining pattern has been shown previously to reflect redistribution of protein from a largely intracellular distribution in normal gut epithelium, to the cell surface in CD [29]. Both the MICA monoclonal antibody and RAET1G1 antiserum showed staining of cells in the lamina propria. Therefore RAET1G1 may have a parallel role to MICA in the pathology of the gut and CD.

### Subcellular localisation of RAET1G1

**Figure 5:**
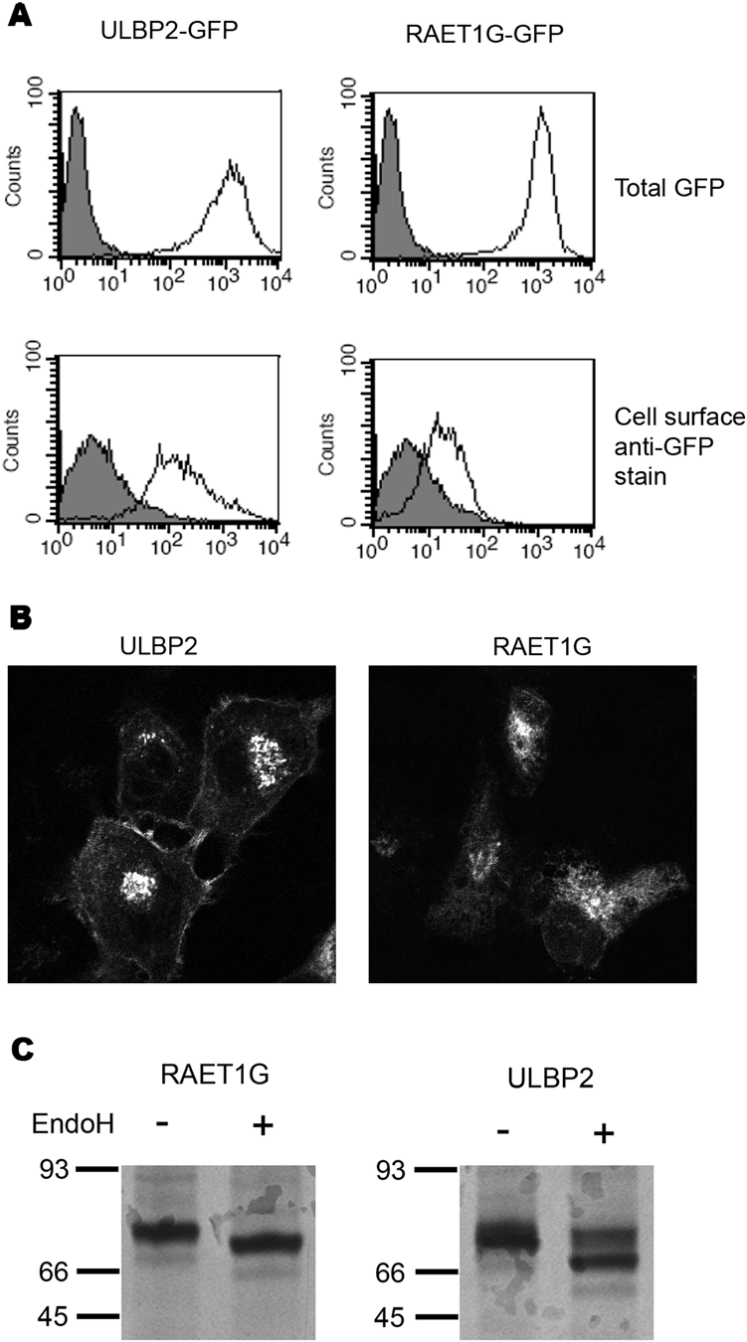
RAET1G1 protein is poorly expressed at the cell surface. (A) Stable cell lines expressing N-terminal GFP fusion proteins of ULBP2, and RAET1G1, were created in HT1080 cells. The transfectants expressed equivalent levels of transgene, as evident by total GFP fluorescence (open histogram), plotted versus untransfected HT1080 cells (gray shaded histogram). Cell surface expression was assessed by staining with an anti-GFP monoclonal antibody followed by an Alexa-Fluor 633 nm (far red) secondary antibody. The RAET1G1 transfectant had only a modest level of staining over untransfected cells, whereas the ULBP2 transfectant stained at a high level. This indicates that a much smaller percentage of RAET1G1-GFP transgene is present at the cell surface when compared to the closely related molecule ULBP2. (B) This observation was confirmed by confocal microscopy. The ULBP2-GFP transfectant showed clearly defined cell surface fluorescence in all cells, whereas cell surface RAET1G1-GFP could not be observed. (C) Stable transfectants were radiolabelled and chased for 180 minutes. Radiolabelled RAET1G1 and ULBP2 were then immunoprecipitated with an anti-GFP antibody. Endo H digests reveal that a substantial proportion of ULBP2 has acquired Endo H resistance after 180 minutes, and hence has trafficked to the cell surface. In contrast RAET1G1 remains Endo H sensitive.

There is evidence for the regulation of the trafficking and subcellular localisation of MICA in a number of different cell types [29], [36], [46]. As RAET1G1 and MICA had highly similar expression patterns in normal and CD gut epithelial cells we went on to examine the subcellular expression of RAET1G further. Stable cell lines of ULBP2 and RAET1G, N-terminally fused to GFP and a Myc-tag, were created in HT1080 cells. By FACS the two cell lines expressed similar amounts of GFP-tagged transgene (Figure 5A). However on staining with an anti-GFP monoclonal antibody less cell surface expression of RAET1G could be detected in contrast to ULBP2 (Figure 5A). Analysing GFP expression by confocal microscopy the ULBP2-GFP transfectants exhibited clear cell surface expression whereas RAET1G-GFP transfectants exhibited much lower levels of staining (Figure 5B).

HT1080 cell lines were radiolabelled, chased for 180 minutes, lysed, and the fusion protein immunoprecipitated with anti-GFP antibodies (Figure 5C). After 180 minutes a substantial proportion of ULBP2-GFP had acquired Endo H resistance, indicating that it had trafficked to the cell surface. In contrast RAET1G-GFP was entirely Endo H sensitive (Figure 5C).

**Figure 6:**
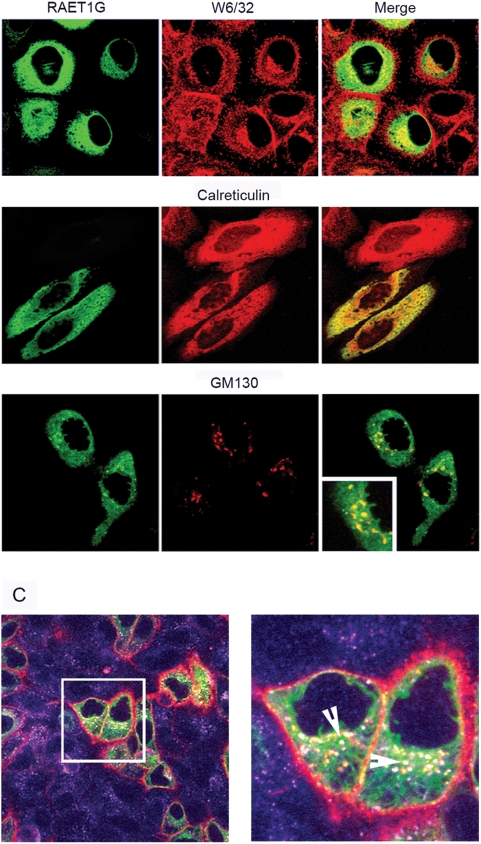
Subcellular localisation of RAET1G1. (A) By confocal microscopy, little RAET1G1 co-localised with MHC Class I (W6/32 antibody) at the cell surface, suggesting that the majority of RAET1G protein is found inside the cell. RAET1G1 does co-localise with calreticulin, a protein expressed predominately in the endoplasmic reticulum (ER). Co-localisation was also seen with the golgi apparatus marker GM130, as highlighted in inset. (C) Antibody internalisation experiment to prove that some RAET1G1 protein does reach the cell surface where it can be internalised into the endocytic pathway. Live cells were incubated with an anti-myc tag antibody prior to fixing and staining. Red represents myc tag staining, purple is staining with an antibody to the early endosome marker EEA1, and green is RAET1G1. Only RAET1G1 positive cells showed staining with the myc antibody; RAET1G1 negative cells only stained with anti-EEA1. In a magnified image EEA1, myc and RAET1G1 co-localised in vesicles (white, marked by arrows) showing that RAET1G1 is being internalised into early endosomes. Also, EEA1 negative, RAET1G1 positive, myc positive vesicles could be seen (yellow), and probably represent other compartments in the endocytic pathway. These data indicate that the vast majority of RAET1G1 protein is present in the ER and early golgi apparatus, and hence does not acquire Endo H resistance. A small minority of RAET1G1 can reach the cell surface, where it can be internalised into the endocytic pathway.

On co-staining with other cellular markers RAET1G showed a contrasting localisation to MHC class I by confocal microscopy, with little co-localisation at the cell surface (Figure 6A). RAET1G mainly co-localised with calreticulin, a protein predominately expressed in the endoplamic reticulum (ER), and the golgi marker GM130 (Figure 6A). An antibody internalisation experiment showed RAET1G protein at the cell surface where it could be internalised into endosomes (Figure 6B).

These data show that whilst the majority of RAET1G protein is found inside the cell, a small proportion does reach the cell surface and can enter the endocytic pathway.

### RAET1G1 protein is expressed in a range of tumours

**Figure 7:**
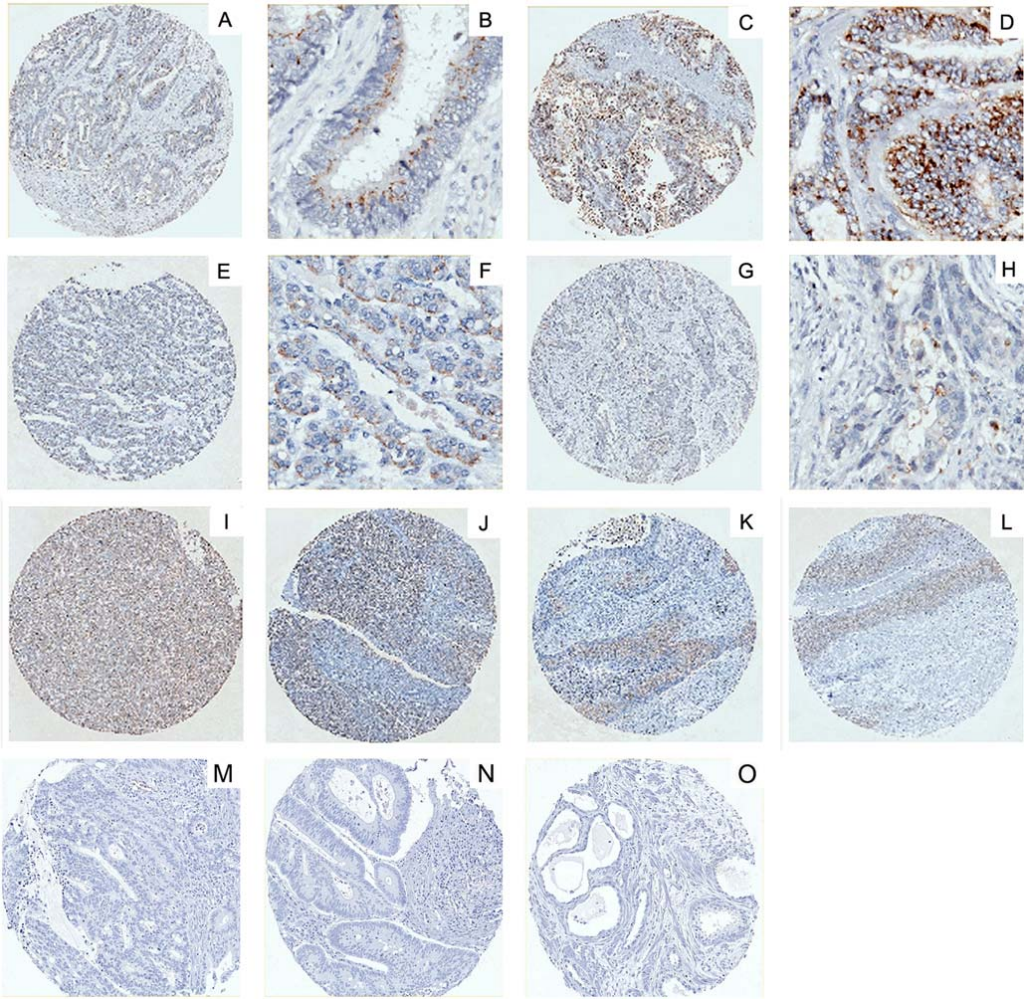
Immunohistochemical localisation of RAET1G in epithelial tumours. A tissue microarray representing 35 different types of primary cancers was probed with the anti-RAET1G antiserum. In contrast normal tissue array widespread staining was observed, for example: (A) and (B) adenocarcinoma of colon, (C) and (D) adenocarcinoma of rectum, (E) and (F) follicular carcinoma of thyroid, (G) and (H) adenocarcinoma of stomach, (I) hepatoma, (J) serous carcinoma of ovary, (K) squamous cell carcinoma of lung and (L) squamous cell carcinoma of skin. All samples of these tumours showed staining, at varying intensity and number of positive cells. Again pre-immune serum did not show staining of tumour sections. (M), (N), and (O) are examples of negative staining in colonic tumours.

We next examined whether RAET1G1 expression was amplified in tumours, using tissue microarray representing 20 different tumour types (Table 1). In contrast to the normal tissue microarray extensive staining was observed in many different cores and was of a higher intensity than in normal tissues (Figure 7). The tumour types stained included; adenocarcinoma of colon, rectum and stomach; squamous cell carcinoma of lung, oesophagus and skin, endometroid carcinoma of uterus, follicular carcinoma of thyroid, hepatoma and serous carcinomas of ovary (Figure 7). Purified pre-immune serum did not stain any core. Therefore RAET1G1 is a novel tumour marker.

### Cellular localisation and function of RAET1G2

**Figure 8:**
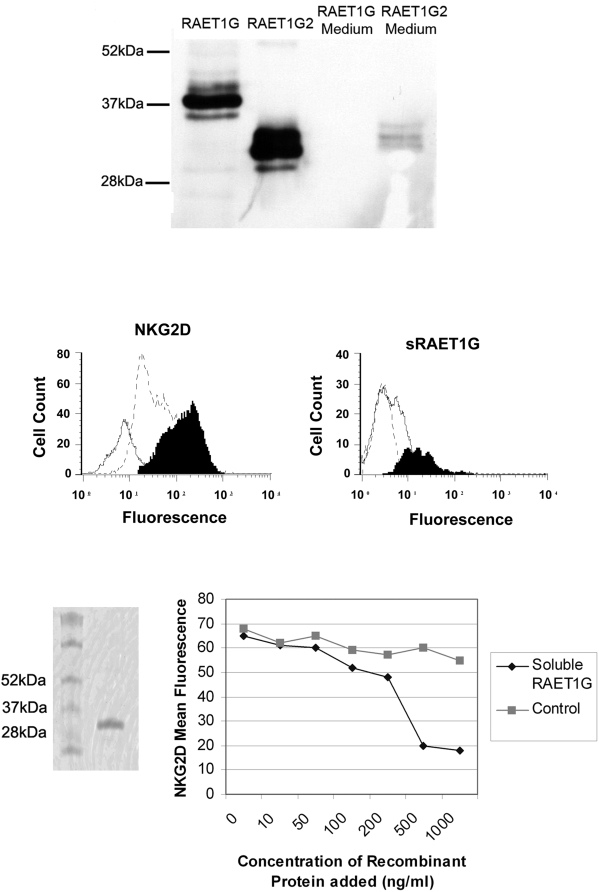
The truncated isoform, RAET1G2, can be secreted by cells and can downregulate NKG2D expression on NK cells. (A) RAET1G2 transcript encodes a protein that is secreted from the cell. C-terminally V5 tagged RAET1G1 and RAET1G2 were transfected into Cos7 cells, RAET1G2 protein was readily detected in the tissue culture media of transfected cells by western blot with an anti-V5 antibody, whereas RAET1G1 was not demonstrated. (B) Soluble RAET1G2 down regulates NKG2D expression on natural killer lymphocytes. Isolated peripheral blood CD3− CD56+ NK cells were incubated for 24 hours at 37°C with culture supernatant from RAET1G1 transfected COS cells (solid black profile) or RAET1G2 transfected COS cells (open dashed profile) and examined for NKG2D expression levels using a PE-labelled anti-NKG2D antibody and flow cytometry. Supernatant from the RAET1G2 transfectant caused a substantial downregulation of NKG2D expression (mean fluorescence intensity = 41.75) compared with supernatant from the RAET1G1 transfectant (mean fluorescence intensity = 144.7). The solid white profile shows background staining with a PE-labelled mouse isotype control (mean fluorescence intensity = 8.1). (C) Soluble RAET1G2 binding to NK cells occurs rapidly but is lost within 24 hours of culture. V5 epitope tagged RAET1G2 was incubated at 37°C with isolated peripheral blood natural killer cells and assayed for binding after 1 hour (solid black profile) and 24 hours (open dashed profile) of incubation. RAET1G2 binding was detected by staining with anti-V5 antibody and PE-labelled goat anti-mouse second stage antibody followed by flow cytometric analysis. The solid white profile depicts background staining with the second stage antibody alone. (D) Coomassie stained SDS-PAGE gel of soluble recombinant RAET1G (rRAET1G). (E) rRAET1G down regulates NKG2D expression. Isolated peripheral blood CD3− CD56+ NK cells were incubated for 24 hours at 37°C with a range of concentrations of rRAET1G. Downregulation of NKG2D was observed at concentrations of rRAET1G as low as 100 ng/ml, but not in the presence of an irrelevant control his-tagged protein.

As RAET1G1 protein is widely expressed in cancer, the possible co-expression of the putative soluble form RAET1G2 may have relevance as a tumour immune evasion mechanism. Both RAET1G1 and RAET1G2 were cloned into a cDNA expression vector with a C-Teminal V5 epitope tag. When transfected into Cos7 cells, RAET1G2, but not RAET1G1, was readily detected in the tissue culture medium by western blot with anti-V5 antibody (Figure 8A). Therefore RAET1G2 is a soluble molecule that can be secreted from cells.

We took cultures of primary NK cells and added the tissue culture medium from Cos7 cells transiently transfected with either RAET1G1 or RAET1G2 for two days. Culturing NK cells with media from RAET1G2 transfectants resulted in a reduction in cell surface NKG2D staining when compared to NK cells cultured with media from RAET1G1 transfectants (Figure 8B). To exclude the possibility that RAET1G2 protein was simply blocking the epitope recognised by the anti-NKG2D antibody we performed a similar control experiment as described previously[24]. Using an antibody to the C-terminal V5 Tag on soluble RAET1G in FACS, NK cells cultured with RAET1G2 containing media for 1 hour showed a slight shift, indicating presence of RAET1G2 protein binding at the cell surface This was not seen in NK cells cultured for 24 hours (Figure 8C). Therefore at the 24 hour time point epitope blocking was not responsible for the observed loss of cell surface NKG2D staining.

To obtain a more quantitative assessment of levels of RAET1G protein required to downregulate NKG2D expression we used recombinant bacterially expressed RAET1G extracellular domain (rRAET1G; Figure 8D). With this reagent we observed downregulation of NKG2D with 100 ng/ml protein after 24 hours incubation (Figure 8E).

## Discussion

NKG2D ligands display considerable diversity in domain structure, and can be expressed independently from each other in cell lines. They are therefore highly unlikely to play functionally identical roles in human disease processes. In order to understand the implications of NKG2D ligand diversity it is necessary to investigate differences, and similarities, in the properties of individual MICA and ULBP/RAET molecules. Here we present the first in depth characterisation of RAET1G1 and RAET1G2.

A systematic search for RAET1G1 protein expression revealed that it has a very restricted expression pattern in healthy tissues. The most striking finding was a staining pattern in the colon and intestinal epithelium similar to that of MICA. Others have previously shown that in the normal gut MICA is found largely inside the cell, however increased expression and/or redistribution of protein to the cell surface may occur in autoimmune conditions such as CD and Crohn's disease[29], [31], [47]. Once at the cell surface MICA can facilitate NKG2D mediated recognition of the gut epithelium and could be a factor in triggering autoimmune attack. Upregulation of cell surface MICA on epithelial cells has been described on binding of a pathogenic *Escherichia coli* protein, AfaE, to CD55[31]. TLR3 signalling has also been shown to induce expression of the mouse NKG2D ligand RAE-1 in the intestinal epithelium[48]. Therefore it is suggested that changes in gut flora may be a trigger for inducing cell surface expression of NKG2D ligands on gut epithelial cells[31], [48]. It has also been shown that some MICA expressing melanoma cell lines do not necessarily express significant levels of MICA at the cell surface[46]. This observation was interpreted as an immune evasion strategy by the cancer, which had switched on a mechanism to retain MICA inside the cell.

Our data show an apparent intracellular staining of RAET1G1 in gut epithelial cells. This distribution was confirmed with a GFP-RAET1G1 fusion protein that was largely intracellularly distributed in the ER and golgi, with only a minority of the protein at the cell surface. It is possible that RAET1G1 could have an as yet undefined function inside the cell. Alternatively it is possible that stimuli exist that can trigger its mobilisation to the cell surface.

RAET1G1 expression was widespread in a range of different epithelial tumour types and is therefore a novel tumour marker. In order to evade immune recognition via NKG2D it has been proposed that tumours release soluble NKG2D ligands from the cell surface[24]. This has the effect of downregulating receptor expression, rather than simply blocking receptor binding to cellular ligand[24]. In the case of MICA and ULBP2 the mechanism for shedding of protein from the cell surface is thought to involve proteolytic cleavage by metalloproteinases[49], [50]. A truncated splicing variant of RAET1G, termed RAET1G2, was widely expressed in primary breast cancer, was secreted from cells, and can downregulate NKG2D expression. This finding has been confirmed in a recent study [51]. Indeed a soluble, secreted splicing variant of RAET1E has also been reported [52] Therefore expression of soluble isoforms of RAET1G and RAET1E could allow cancer cells to produce soluble NKG2D ligand without the need for metalloproteinase activity. Demonstrating the presence of soluble RAET1G2 protein in cancer patients is the obvious next step, however it will be technically challenging to produce specific antibodies as RAET1G2 is 95% identical to the ULBP2 molecule. In addition RAET1G has a highly similar promoter to ULBP2 and in all 20 cell lines screened in this study RAET1G splicing variants and ULBP2 were co-expressed (data not shown).

Examination of RAET1G1 and RAET1G2 expression in clinically defined sample sets of different cancers will be crucial to assess the diagnostic/prognostic potential of the molecules. RAET1G also represents a potential avenue for cancer therapy, for example by inducing its expression on NKG2D ligand negative tumours or as a target for anti-cancer monoclonal antibodies.
