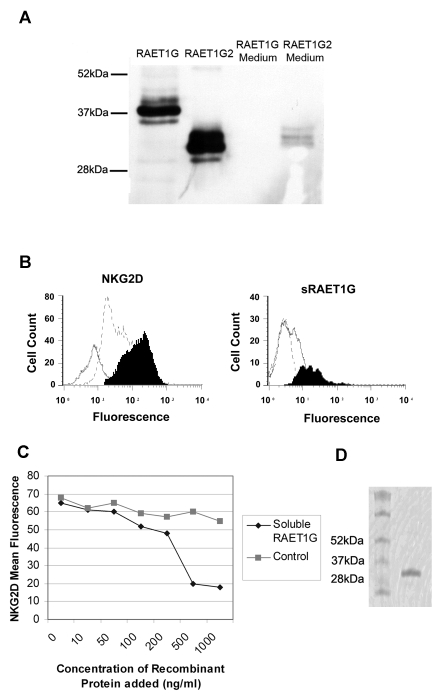# Correction: Cellular Expression, Trafficking, and Function of Two Isoforms of Human ULBP5/RAET1G

**DOI:** 10.1371/annotation/8501cd33-6c9e-437c-9ea1-f96fbb4140d6

**Published:** 2009-03-25

**Authors:** Robert A. Eagle, Gillian Flack, Anthony Warford, Jesús Martínez-Borra, Insiya Jafferji, James A. Traherne, Maki Ohashi, Louise H. Boyle, Alexander D. Barrow, Sophie Caillat-Zucman, Neil T. Young, John Trowsdale

The subheadings on figure 8 are incorrect. The correct figure can be viewed here: 

**Figure:**